# Dynamic analysis of soil erosion in the affected area of the lower Yellow River based on RUSLE model

**DOI:** 10.1016/j.heliyon.2023.e23819

**Published:** 2023-12-20

**Authors:** Ying Zhang, Pengyan Zhang, Zhenyue Liu, Guangrui Xing, Zhuo Chen, Yinghui Chang, Qianxu Wang

**Affiliations:** a School of Urban Economics and Public Administration, Capital University of Economics and Business, Beijing, 100070, China; b College of Geography and Environmental Science, Henan University, Kaifeng, 475004, China; c Xinyang Vocational and Technical College, Xinyang, 464000, China; d School of Medicine, Case Western Reserve University, Cleveland, OH, 44106, USA

**Keywords:** Soil erosion, RUSLE, Geodetector, The affected area of the lower Yellow river

## Abstract

With the accelerated development of urbanization, the exploration and usage of land resources is becoming more and more frequent, which leads to the decline of soil quality, resulting in a series of soil ecological issues, such as soil nutrient loss, soil quality degradation and destruction. At present, the contradiction between soil erosion and sustainable development of human society has become one of the hot issues studied by scholars. The Yellow River Basin is an important experimental area for high-quality development in China, constructing the Yellow River Ecological Economic Belt play an important role in China’s regional coordinated development. Although most of the affected area of the Lower Yellow River (AALYR) is in the plain, they have a large population density and are in the historical farming area. In latest years, because of the development and transformation of modern society, their ecological environment has become more fragile and soil erosion problems has become increasingly serious. Studying and analyzing soil erosion is of vital meaning for ecological protection and can provide scientific support for soil conservation work. Depending on the data of precipitation, soil properties, land use, population, etc., this paper studies and analyzes the soil erosion in AALYR from 2000 to 2020 through the RUSLE. We found that during the 20 years the proportion of very slight and slight grade area increased, and the distribution of moderate and above erosion grade was less, mainly in Zibo, Jinan, Anyang, Zhengzhou, and Tai 'an. Nearly three quarters of the regional soil erosion grade didn’t change, apart from the increase of slight grade area, the other erosion grades area showed a downward trend. We take the city, county and town zoning analysis find that as the scale decreases, the area of serious erosion grades increases, and the distribution is gradually detailed. Land use is the main influencing factor of erosion except DEM. Forestland and grassland are larger of the soil erosion in various types of land use. Through these conclusions in this paper, it is promising to provide theoretical references for the ecological environment governance and high-quality and sustainable development of great river basins of the world and similar regions.

## 1 Introduction

Soil erosion is generally viewed to be the result of a confluence of natural factors and human activities [1]. With global warming, extreme rainfall and severe weather are becoming more and more frequent. Extreme rainfall events lead to soil nutrient depletion and destruction and loss of land productivity [2], which strongly controls the amount of soil erosion every year and for many years [3]. At the same time, increasing human activities such as population increase, accelerated urbanization and land use changes, have accelerated soil degradation and caused a decline in regional soil quality [4]. Soil erosion has gravely threatened the social and economic security of various countries in the world [5], its governance has received extensive attention from scholars around the world [6]. Soil erosion not only leads to soil degradation, resulting in a decline in land productivity, but also leads to massive soil erosion, it can easily lead to ecological problems such as river siltation and increased floods, thus threatening the development of human society [7]. Based on this, quantitative analysis of soil erosion is essential for controlling soil erosion, implementing the overall requirements of ecological civilization construction, and achieving sustainable social and economic development.

For the research of soil erosion, scholars engaged in this work have passed a lot of experiments, combined with statistics and analysis, among which the USLE (Universal Soil Loss Equation) [8] and WEPP [9] models of the United States have been recognized by many scholars. As early as 1882, the German soil scientist Ewald Wollny established the runoff plot method and began the quantitative research of soil erosion [10]. After years of development, Wischmeier and Smith [11] formulated the USLE through artificial simulated rainfall test and observation data analysis of multiple runoff plots. Later, after a lot of practice by experts and scholars, in 1986, the USDA developed a water erosion prediction model (WEPP) [12] that comprehensively considered the hydrological conditions of the land. In 1997, a modified equation (Revised Universal Soil Loss Equation, RUSLE) was obtained which can carry out soil erosion prediction of single rainfall. And in 2002, Liu et al. [13] analyzed the runoff plot data, established the CSLE (China’s Soil Loss Equation). Scholars’ early studies on soil erosion models are mainly the introduction and improvement of models such as WEPP, USLE, and others. Later research is based on the USLE model. Through a series of exploration and practice of soil erosion impact factors [14], the research which are based on the USLE/RUSLE model has many practical applications in regions and basins.

At present, scholars’ research on soil erosion mainly covers several aspects such as scale, method, content etc. In terms of scale, there are mainly natural regional scales such as watershed [15,16], plateaus [[17], [18], [19]], basins [20], and administrative unit scales such as countries [21], provinces [22], cities [23], soil erosion zoning. Based on these research scales, scholars have made more estimates of soil erosion in Chinese watersheds, but relatively few studies on soil erosion at the scale of the lower reach of the Yellow River (LYR), and most of them take administrative units [24] or typical geomorphological areas [25] as research objects. There are few reports on soil erosion estimation in LYR by USLE/RUSLE. From the perspective of methods, there are mainly USLE/RUSLE model estimation [26], InVEST (Integrated Valuation of Ecosystem Servicesand Tradeoffs) model analysis [27], WaTEM/SEDEM (Water and Tillage Erosion Model/Sediment D Elivery Model) model evaluation [16]. On the whole, the basic trend and method of quantitative evaluation of soil erosion are supported by GIS and RS, using soil erosion models such as USLE/RUSLE, WEPP, CSLE, and others, to create a soil erosion impact factor library to complete the quantitative evaluation of soil erosion. Among them, WEPP model belongs to physical process model, and its applicability in AALYR is still unknown. The RUSLE method comprehensively considers the effects of rainfall, soil, terrain, vegetation and engineering measures. Compared with the USLE model, it is suitable for more complex terrain and has strong applicability and is widely used in quantitative soil erosion studies around the world [25,28]. From the perspective of research content, it is mainly related to climate change [29], land use [16], mining area development [30] and other factors. There are also studies on a single comprehensive understanding and analysis of soil erosion, and to investigate the impact mechanisms behind it [31].

**Fig. 1:**
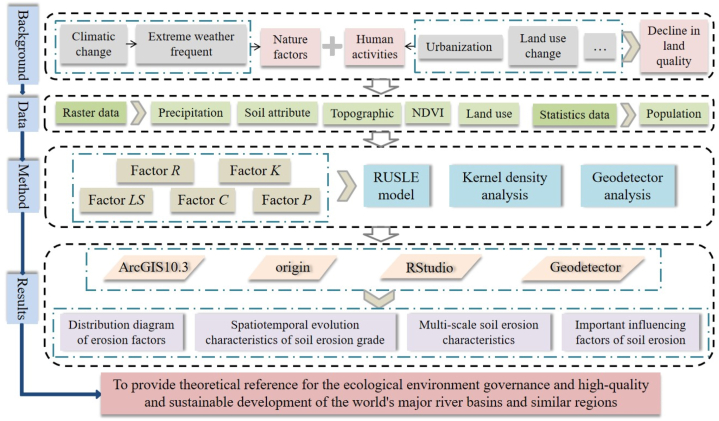
Research framework.

As the birthplace of China’s agricultural civilization, the Yellow River Basin (YRB) has a long history of agricultural development. It has been in high-intensity development for a long time, resulting in contradictions such as fragile ecological environment, water shortage and prominent water environment problems in YRB [32]. As one of the China’ main grain producing areas and core areas, AALYR belongs to the traditional agricultural area. It is one of the typical regions in China with rapid economic development and greater impact from human activities [33,34]. While facing the transformation to a modern society, it also bears the pressure of dense population and the resource and environmental load problems brought by agricultural development [35]. On the basis of these, the development of urbanization and cultivated land rotation have brought many environmental problems to AALYR. With the increasing demand for limited soil resources and the gradual reduction of per capita cultivated land, soil erosion and destruction has become an important issue affecting the ecological protection and sustainable economic and social development in AALYR. The quantitative research of soil erosion in AALYR has become an urgent problem to be solved. Therefore, based on the data of remote sensing image, land use, soil properties and so on, combined with RUSLE model and GIS technology, this paper maps and analyzes the soil erosion assessment in AALYR, so as to have a comprehensive and holistic understanding of its soil erosion situation. It can be used as the basis for developing AALYR soil protection measures, and also contributes to the ecological environment management, high quality and sustainable development of the world’s great river basins (Fig. 1).

## 2 Materials and methods

### 2.1 Study area

**Fig. 2:**
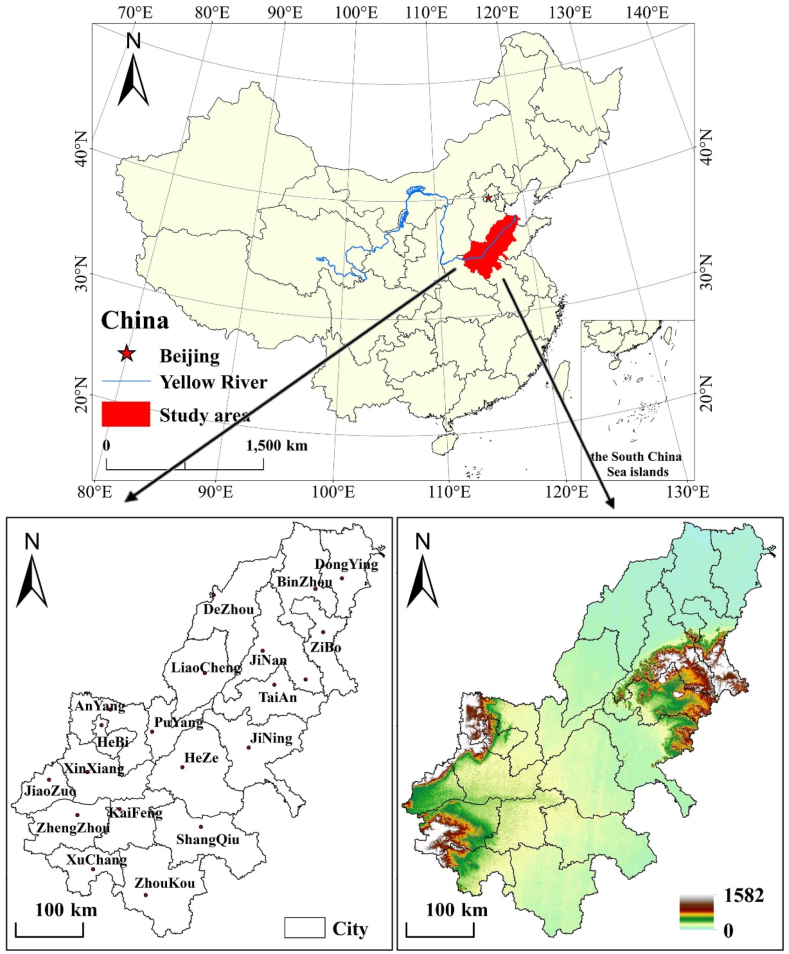
Study area.

Considering the ancient Yellow River flood distribution, and reference to relevant literature [36,37], 20 prefecture-level cities of Henan and Shandong provinces were selected as AALYR. Among them, Laiwu was assigned to Jinan in 2019. In this paper, it is attributed to Jinan for analysis. The overview of study area is shown in Fig. 2. The topography of AALYR is dominated by plains, hills and fan delta. The terrain is undulating and gentle, the sediment is constantly silting up, and the downstream river channel is rising year by year, thus forming the world’s famous “suspended river”. Under the national soil and water conservation zoning, AALYR is located in the China’ Northern Rocky Mountain area [38]. The soil is mainly fluvo-aquic soil, frequent cultivate leads to weak corrosion resistance and high risk of soil erosion.

### 2.2 Data sources

The relevant data required are as follows (Table 1).

**Table 1:** Data declaration.

Data	Year	Precision	Source	Usage
Precipitation [39]	2000, 2005, 2010, 2015, 2020	1 km	http://data.tpdc.ac.cn/	*R*
Soil properties	–	1 km	http://www.issas.ac.cn/kxcb/zgtrxxxt/	*K*
DEM	–	30 m	http://www.gscloud.cn/search	*LS*
NDVI	2000, 2005, 2010, 2015, 2020	250 m	https://www.nasa.gov/	F, *C*
Land use	2000, 2005, 2010, 2015, 2020	1 km	https://www.resdc.cn/Default.aspx	*P*
Population	2020	1 km	https://landscan.ornl.gov/	Influencing factors

### 2.3 Methods

#### 2.3.1 RUSLE model

RUSLE is an effective tool for quantitative estimation of soil erosion [40] is widely used. In this paper, according to the RUSLE model method, the results of spatial distribution of *R*, *K*, *LS*, *C*, and *P* factor are obtained. Then, the factors are multiplied in ArcGIS 10.3 to obtain the estimation results. Calculation formula is as follows (1):

(1) A=R×K×LS×C×P

where: *R* is rainfall erosivity factor, *K* is soil erodibility factor; *LS* is slope length factor; *C* is vegetation cover management factor; *P* is the factor of soil and water conservation measures; *A* is the amount of soil erosion t/(hm^2^·a), the unit is converted to t/(km^2^·a) after multiplying 100.

##### 2.3.1.1 Factor *R*

Rainfall is the direct external driving force of soil erosion. *R* reflects the influence of rainfall on soil erosion. Considering the size of AALYR, the availability of data and the simplicity of processing, this paper chooses Wischmeier [41] formula to obtain *R*. The formula is as follows (2):

(2) $$R=\sum_{j=1}^{12}1.735\times 10^{\left[\left(1.5\times \mathrm{lg}\frac{p_{j}^{2}}{p}\right)-0.8188\right]}$$

where: *R* is the rainfall erosivity factor; *p*_*j*_ is monthly rainfall; *p* is the annual rainfall.

##### 2.3.1.2 Factor *K*

*K* is determined by the physical properties of the soil, but it is usually difficult to obtain parameters such as soil structure and soil infiltration ratio [42]. In view of the easy availability of data, the EPIC (Erosion-Productivity Impact Calculator) model constructed by Williams [43] is selected to calculate the *K* factor, which responded well to soil erodibility. The formulas are as follows (3) and (4):

(3) $$K=\left\{0.2+0.3\;\exp\left[-0.0256SAN\left(1-\frac{SIL}{100}\right)\right]\right\}\times {\left(\frac{SIL}{CAL+SIL}\right)}^{0.3}\times \left[1-\frac{0.25OC}{C+\exp\left(3.72-2.95OC\right)}\right]\times \left[1-\frac{0.7SNI}{SNI+\exp\left(-5.51+22.9SNI\right)}\right]$$

(4) $$SNI=1-\frac{SAN}{100}$$

where: *SAN* is the percentage of sand (%); *SIL* (0.002∼0.05 mm) is the percentage of silt (%); *CAL* is the content of clay (%) [44] (See supplementary file for details); *OC* is the organic carbon content (%).

##### 2.3.1.3 Factor *LS*

*LS* is obtained based on the *LS* factor calculation tool [45,46] established by Fu et al. [47] for evaluating soil erosion in China. The formulas are as follows (5), (6), and (7):

(5) $$L_{i}=\left(\lambda_{out}^{m+1}-\lambda_{in}^{m+1}\right)/\left[\left(\lambda_{out}-\lambda_{in}\right)22.13^{m}\right]$$

(6) $$\begin{array}{l} m=0.2,\theta <0.5{}^{\circ}\\ m=0.3,0.5{}^{\circ}\leq \theta <1.5{}^{\circ}\\ m=0.4,1.5{}^{\circ}\leq \theta <3{}^{\circ}\\ m=0.5,\theta \geq 3{}^{\circ} \end{array}$$

(7) $$\begin{array}{l} S=10.8\;\sin\;\theta +0.03,\theta <5{}^{\circ}\\ S=16.8\;\sin\;\theta -0.5，5{}^{\circ}\leq \theta <10{}^{\circ}\\ S=21.91\;\sin\;\theta -0.96,\theta \geq 10{}^{\circ} \end{array}$$

where: *L*_*i*_ is slope length factor of *i* th grid; $\lambda_{out}$, $\lambda_{in}$ is slope length of the grid outlet and inlet respectively; *m* is slope length index.

##### 2.3.1.4 Factor *C*

The higher the *C*, the worse the vegetation cover [48]. According to the Rocky Mountain area of Northern China where the study area is located and referring to scholars [49,50] used the estimation method model in the calculation of China’ soil erosion to calculate *C* factor. The formulas are as follows (8) and (9):

(8) $$F=\left(NDVI-NDVI_{\min}\right)/\left(NDVI_{max}-NDVI_{min}\right)$$

(9) $$\begin{array}{l} C=1,F=0\\ C=0.6508-0.3436\times \text{lo}g_{10}F,0<F\leq 78.3\%\\ C=0,F＞78.3\% \end{array}$$

where: *F* represents fractional vegetation cover.

##### 2.3.1.5 Factor *P*

The higher the *P*, the greater the possibility of soil loss (its value is between 0 and 1) [51]. The study area is in the rocky mountain, so the *P* factor are assigned by relevant literatures of the same type of research area [52,53], as shown in Table 2.

**Table 2:** *P* factor value.

Land use	Dry land	Paddy field	Forest land	Shrub land	Sparse woodland
*P*	0.3	0.01	1	0.2	0.2
Land use	Other forest land	Grassland	Water area	Built-up land	Unused land
*P*	0.4	0.2	0	1	1

#### 2.3.2 Kernel density analysis

Kernel Density Analysis will produce a density surface to reflect the aggregation of the elements [54]. Generally speaking, kernel density analysis refers to the probability of geographical events occurring in different spatial locations. The probability of point-intensive regional events is high, and its density is large; on the contrary, the events in the region of sparse points have a low probability of occurrence and a small density. Through kernel density analysis, we can intuitively see the probability of soil erosion, reflecting its aggregation characteristics. Calculation formula is as follows (10):

(10) $$f\left(x\right)=\frac{1}{nh^{d}}\sum_{g=1}^{n}k\left(\frac{x-x_{g}}{h}\right)$$

where: *f(x)* is kernel density value, *k* is kernel function, *x*_*g*_ represents the independent distribution of *g* sample points, and *g* is the number of points in the sample range.

#### 2.3.3 Multi-scale analysis

**Fig. 3:**
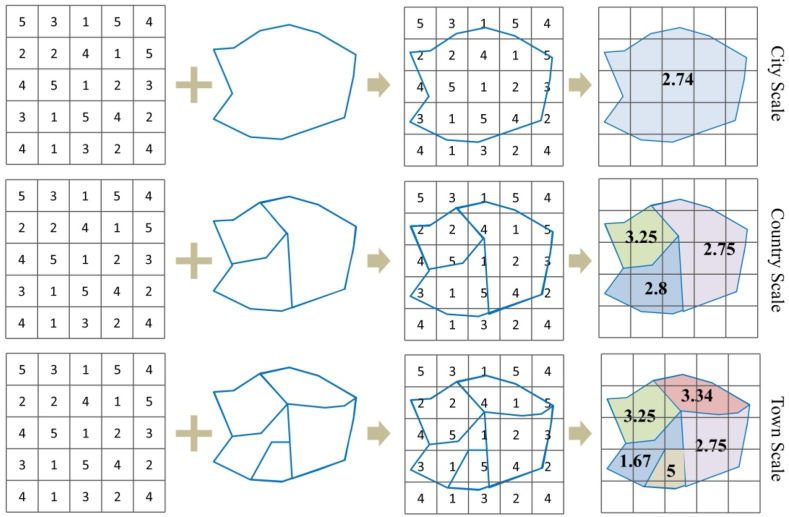
Multi-scale analysis diagram.

In this paper, through the administrative vector data of the city, county and town, the soil erosion situation in 2000, 2005, 2010, 2015 and 2020 is counted to analyze the soil erosion characteristics at different scales. The average value of all pixels in the grid belonging to the same area as the output pixel is calculated, so as to measure the average soil erosion amount of each administrative region at different scales. According to the principle of zoning statistics, in the larger administrative divisions, the same amount of soil erosion, due to the larger coverage area, the average treatment compared with the smaller administrative grading changes are not obvious. Therefore, the smaller the scale is, the more prominent the serious erosion area is. In this way, according to the erosion in the administrative district, relevant policy measures for the avoid and contain of soil erosion can be effectively formulated and implemented by administrative means, which is more conducive to the management of regional soil erosion. The brief method is illustrated in Fig. 3.

#### 2.3.4 Geodetector

Geodetector is a statistical analysis method based on spatial differentiation theory [55]. It can reflect the similarity of the same region and the difference of different regions, so as to obtain the spatially distinctive features of geographical phenomena and the driving factors behind them [56]. The Geodetector includes four modules: factor detection, interactive detection etc. The two main detections used are as follows.

(1) The factor detection reflects the ability of a single factor to explain a geographic phenomenon. Formula is:

(11) $$q=1-\frac{\sum_{e=1}^{l}N_{e}\sigma_{e}^{2}}{N\sigma^{2}}$$

where: *l* represents the number of layers of the independent variable X; *N*_*e*_ and *N* are the number of samples in the layer and region; σ^2^ and $\sigma_{e}^{2}$ are the overall variance of the sample. When σ^2^≠0, the model is established. *q* ∈ [0,1], it reflects the explanatory power of X for the spatially differentiated characteristics of soil erosion.

(2) The interactive detection reflects the common explanatory power of the interaction between factors. Based on the analysis results, the important influencing factors of soil erosion can be understood.

## 3 Results

### 3.1 Spatiotemporal characteristics of soil erosion in AALYR

#### 3.1.1 Spatial pattern of soil erosion factors

RUSLE for assessing soil erosion is composed of *R*, *K*, *LS*, *C*, and *P* factors. *R* is mainly caused by heavy rainfall, reflecting the influence of rainfall. Factor *K* reflects soil erodibility and soil erosion resistance. *LS* reflects the influence of topographic features. Factor *C* describes the effect of vegetation coverage on soil erosion. *P* mainly reflects the influence of agricultural tillage measures, engineering measures and plant measures. They can be obtained according to the calculation method in the RUSLE model.

**Fig. 4:**
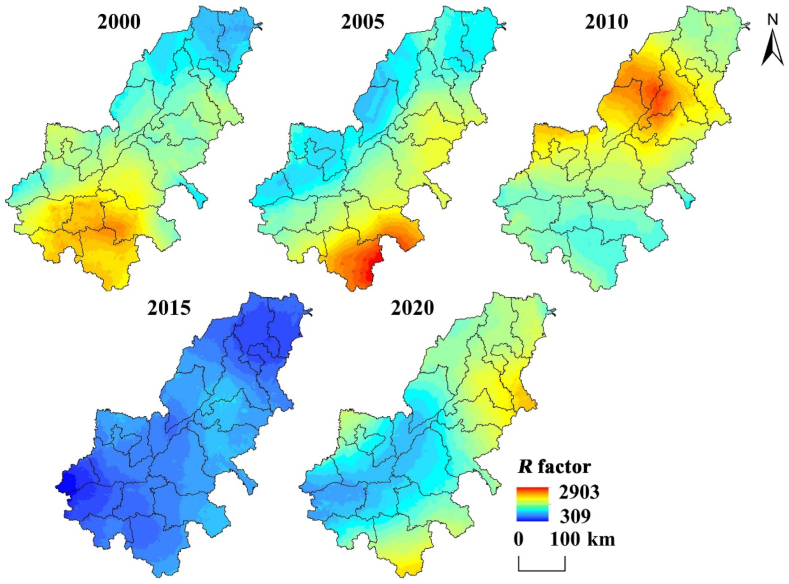
*R* factor distribution map of AALYR.

*R* factor is an important influencing factor in this paper. *R* is obtained from Formula (2) (Fig. 4). From Fig. 4, the high value of *R* factor is mostly distributed in or near the provincial capitals of Zhengzhou and Jinan in the five years. This may be due to the large population and intensive industry in large cities, which triggers the urban rain island effect and leads to an increase in precipitation. In addition, the high degree of hardening of the ground will affect the infiltration process of rainwater, which will lead to the increase of surface runoff in these areas and cause soil erosion. The *R* factor was generally lower in 2015, due to the impact of drought, and the precipitation was lower than other years [57]. In general, due to the effect of various factors, the spatial difference of *R* factor in different years is obvious, and it also has different influence on soil erosion in that year.

**Fig. 5:**
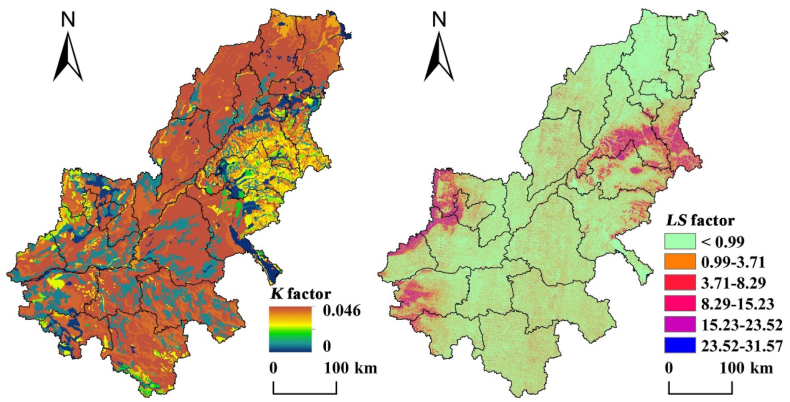
*K* and *LS* factor distribution map of AALYR.

According to the RUSLE model, the factor *K* and *LS* are obtained (Fig. 5). From Fig. 5, the *K* factor shows a high value in most areas, and the *K* factor in a few and very few areas show medium and low values, mainly distributed in the eastern. This is largely related to the large-scale distribution of fluvo-aquic soil in AALYR, its physical and chemical properties determine the risk of soil erodibility. *LS* is mainly related to the terrain, the value of high altitude and large slope is larger, so it is higher in the mountains of central-south Shandong province, Taihang Mountains and Songshan Mountains in the study area, and the value in other areas is lower. Among them, the mountains of central-south Shandong province, Taihang Mountains and Songshan Mountains are in the eastern, western and southwestern.

**Fig. 6:**
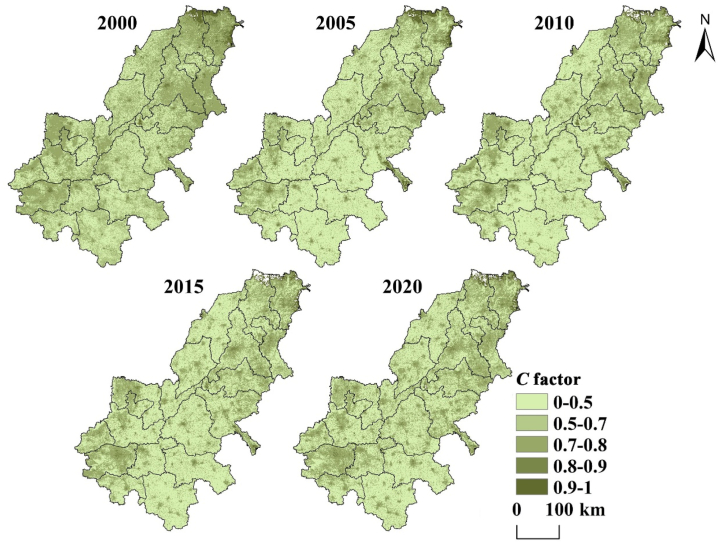
*C* factor distribution map of AALYR.

Factor *C* is calculated by the RUSLE model is shown in Fig. 6. In areas with dense vegetation, the water retention effect of plant canopy making soil erosion lighter. On the whole, the high value of *C* factor is mainly in the north and scattered in the middle, corresponding to the waters and some bare areas in the study area. In addition, the implementation of returning farmland to forest policies around 2000 has greatly increased the vegetation coverage. Fig. 6 shows that factor *C* is changing in a good direction from 2000 to 2020, which is conducive to preventing soil erosion.

**Fig. 7:**
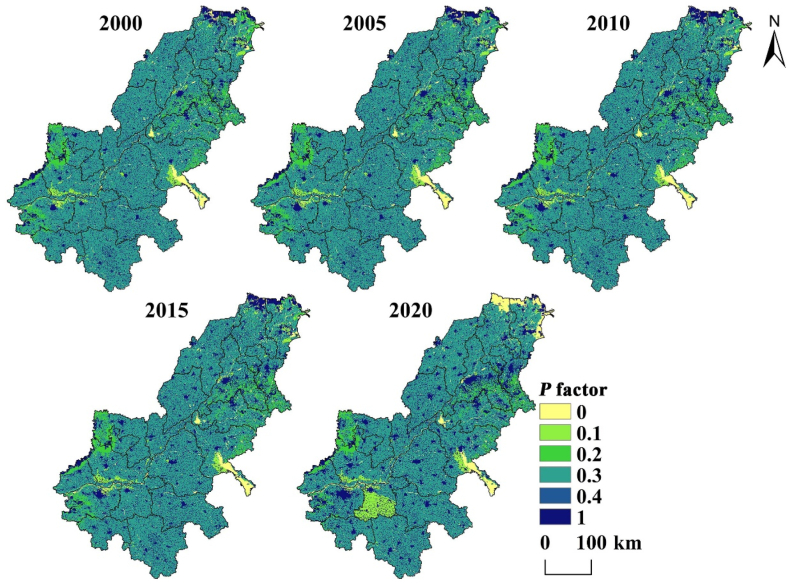
*P* factor distribution map of AALYR.

Factor *P* is an important factor affecting soil erosion. Factor *P* results are obtained according to Table 2 (Fig. 7). According to the distribution of *P* (Fig. 7) and its calculation method, the *P* of unused land and built-up land is 1, so they are the high-value concentration areas of the *P*. Correspondingly, the low values are mainly distributed in the waters. *P* value is closely related to changes in the land use.

#### 3.1.2 Spatial distribution characteristics of soil erosion grade

According to the results of each factor obtained in Section 3.1, after being converted into a unified resolution (30 m), the soil erosion from 2000 to 2020 is obtained in ArcGIS 10.3 through map algebra tool. To more clearly analyze the changes of erosion from 2000 to 2020, we classify erosion grades according to the classification standard [58] (Table 3).

**Table 3:** Classification standard.

Grade	Soil erosion (t/(km^2^·a))
Very slight	≤500
Slight	(500,2500]
Moderate	(2500,5000]
Severe	(5000,8000]
Very severe	(8000,15,000]
Extremely severe	≥15,000

**Fig. 8:**
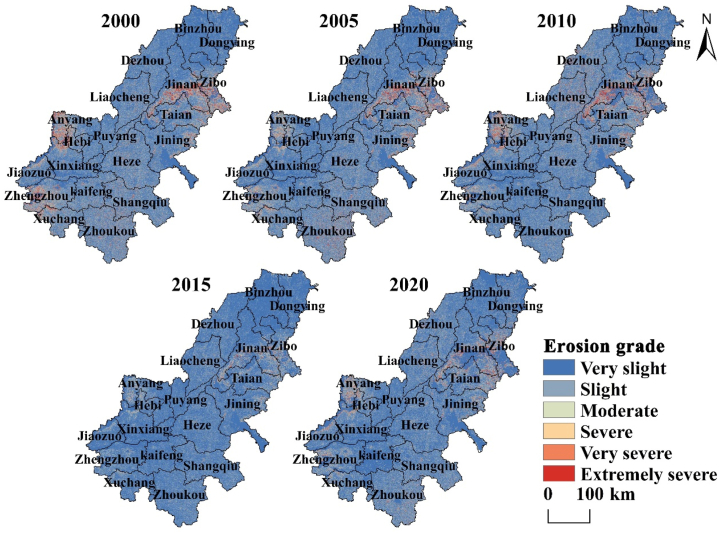
Grade distribution map of erosion intensity in AALYR.

According to the erosion grade map (Fig. 8), the distribution pattern of erosion grade is generally consistent. Very slight grade area is the largest, followed by slight and moderate grade, which occupy most area. Severe, very severe and extremely severe grade is less distributed, mainly in Jinan, Zibo, Tai 'an, Anyang, Hebi, and Zhengzhou.

**Fig. 9:**
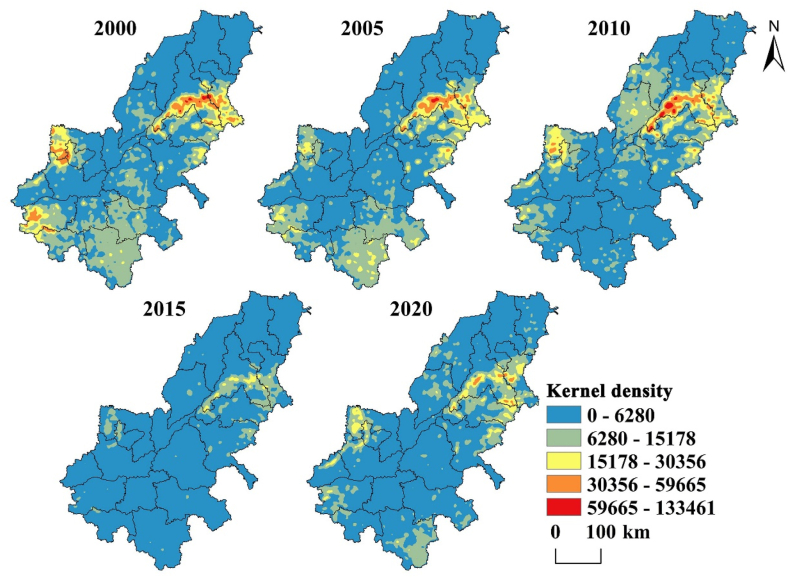
Kernel density analysis map of soil erosion in AALYR.

To intuitively explore the distribution of serious erosion area, using ArcGIS 10.3 kernel density analysis tool, the amount of soil erosion at each point was used as the basic data for analysis, and the kernel density map is obtained (Fig. 9). From Fig. 9, we can find that the areas with high erosion grade are obviously distributed in the western, southwestern, and eastern, and some years are also more serious in the south. The soil erosion in 2015 was the weakest compared with other years, which was largely due to the low rainfall erosivity in 2015 and didn’t have much impact on soil erosion, while the rainfall erosivity in other years was relatively high. Generally, the density center of the serious erosion region is gradually weakening, reflecting the gradual improvement of the soil and water environment.

To reveal the characteristics of erosion grade changes, a statistical table of soil erosion grade change area is made (Table 4). From Table 4, the soil erosion grade in the 110.64 × 10^3^ km^2^ area of the study area don’t change, and the area accounted for 74.94%. The erosion grade of 16.37% of the area decreased, and the proportion of one grade decreased was the largest in the area of 24.17 × 10^3^ km^2^, which was 10.12%. There are 1.12 % of the five grades of decline, while only 0.07 % of the five grades of increase. The erosion of these areas with large grade decline was more serious in 2000 and improved by 2020. The area with increased erosion grade accounted for 8.69%, which was less than the area with decreased erosion grade. The area of 6.39 % increased by one grade, and the increase of most erosion grades was relatively small.

**Table 4:** Statistics of erosion grade change area in study area.

Grade change	−5	−4	−3	−2	−1	0	1	2	3	4	5
Area (10^3^ km^2^)	1.66	1.47	1.88	4.23	14.95	110.64	9.44	2.16	0.88	0.25	0.11
Proportion (%)	1.12%	0.99%	1.27%	2.86%	10.12%	74.94%	6.39%	1.46%	0.60%	0.17%	0.07%

Note: 0 represents that the erosion intensity grade has not changed; the positive number represents the increase of erosion intensity grade, and the greater the value, the more prominent the degree of strength deterioration. The negative number represents the decrease of erosion intensity grade.

#### 3.1.3 Spatiotemporal evolution characteristics of soil erosion grade

**Fig. 10:**
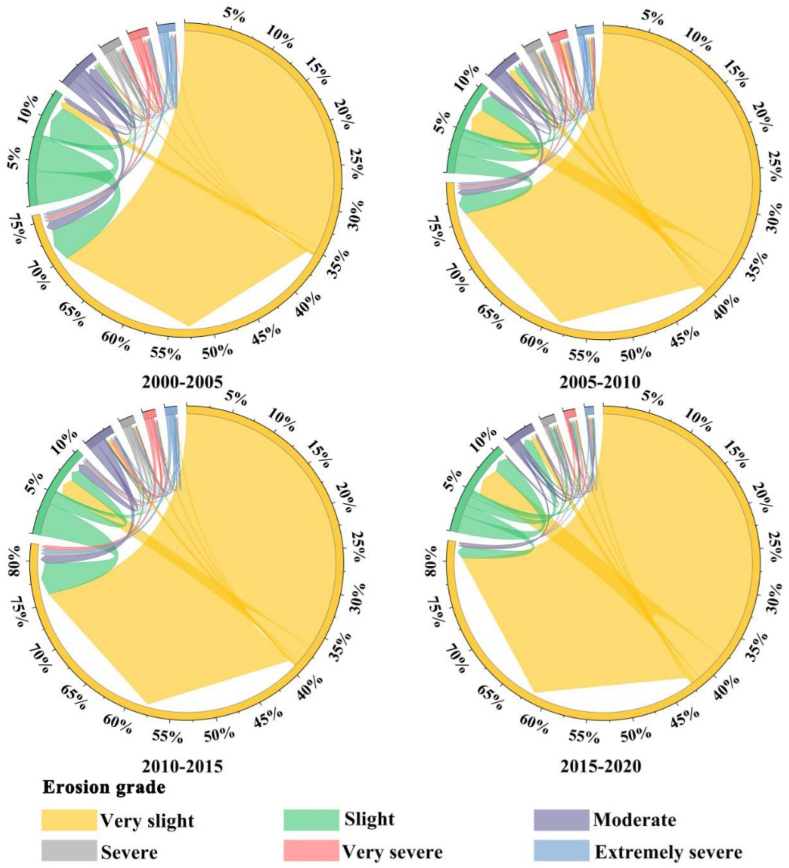
Conversion map of erosion grade in AALYR.

According to the erosion during study period, obtains the transformation map of soil erosion grade (Fig. 10). The width between the chord lengths at both ends in Fig. 10 represents the proportion of the transfer area, and the arrow represents the direction of the transfer. From Fig. 10, the very slight and slight erosion in each period occupied most of the area, all of which were above 85%, and up to 95.20% in 2015. According to the transformation between different erosion grades shown in Fig. 10, we can see that during the study period, in the 2000–2005 stage, the area of slight to very slight was the largest, accounting for 7.58%, followed by the 2010–2015 stage, very slight to slight with an area ratio of 7.40 %. In addition, compared with the transformation of erosion grade in different time periods in Fig. 10, it can be found that some variations in the shift of soil erosion grades at each stage. In addition to the erosion grade unchanged, the transition of erosion grade from very slight to slight, and from slight to very slight occupies a relatively large area. On the whole, the area proportion of very slight in the study area increased from 71.04 % to 79.58%, with an increase of 8.54%. The area proportion of other erosion intensity grades decreased, with a decrease of 0.5%–5%. The largest decrease was slight erosion, which decreased by 4.83%.

According to the soil erosion, the statistical table of erosion grade area from 2000 to 2020 is obtained (Table 5). It can be seen from Table 5 that the extremely severe area reached the maximum in 2000 and the minimum in 2015. The very slight erosion area reached the maximum in 2015, which was 125.78 × 10^3^ km^2^, and the minimum in 2000, reflecting that the erosion situation is the best in 2015. In addition, the area of very slight and slight erosion increased from 128.91 × 10^3^ km^2^ in 2000 to 133.93 × 10^3^ km^2^ in 2020. On the basis of the increase of low erosion grades area, the area of the more severely eroded area shows a decrease. Severe, very severe and extremely severe erosion grade decreased to 7.79 × 10^3^ km^2^ by 2020.

**Table 5:** Area of each erosion grade in AALYR from 2000 to 2020 (10^3^ km^2^).

Grade	2000	2005	2010	2015	2020
Very slight	105.32	120.03	116.48	125.78	117.55
Slight	23.59	15.34	15.72	15.08	16.38
Moderate	8.11	5.34	6.14	3.71	6.00
Severe	3.95	2.75	3.06	1.66	2.92
Very severe	3.84	2.74	3.12	1.07	2.70
Extremely severe	3.45	2.44	3.22	0.66	2.17

### 3.2 Soil erosion characteristics at different scales

**Fig. 11:**
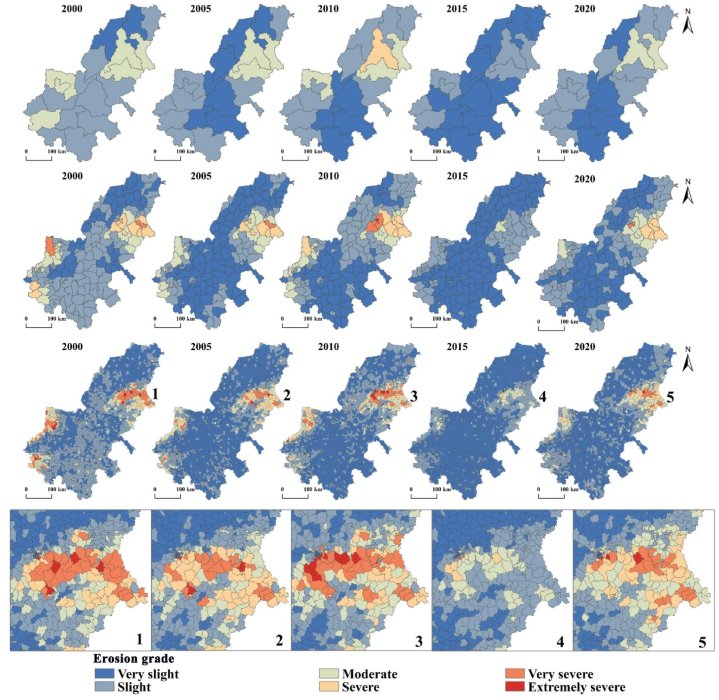
Soil erosion intensity grades at different scales in AALYR (upper to lower: city, county, town).

In order to further understand the soil erosion situation in AALYR from an administrative perspective, the soil erosion characteristics of AALYR are analyzed from multiple scales of city, county and town. Table 6 shows the area of erosion grades at different scales of AALYR. Fig. 11 shows the soil erosion grade at different scales during the study period. From Table 6, the erosion grade is mostly distributed in very slight, slight and moderate grade on the city scale. The largest area is the very slight erosion in 2015, the smallest area is the severe erosion in 2010, which is 10.231 × 10^3^ km^2^. At the county scale, the distribution of erosion grade increased to extremely severe erosion grade. At the town scale, each erosion grade is distributed, but mostly concentrated in the lower erosion grade. The largest area is 112.505 × 10^3^ km^2^, which is the very slight erosion in 2015, and the smallest area is the extremely severe erosion in 2015.

**Table 6:** Each erosion grade area at different scales in AALYR (10^3^ km^2^).

		Very slight	Slight	Moderate	Severe	Very severe	Extremely severe
2000	City	19.380	89.531	41.020	–	–	–
	County	40.030	81.443	15.103	10.597	2.758	–
	Town	54.759	71.266	9.641	6.548	6.218	1.006
2005	City	61.361	64.637	23.933	–	–	–
	County	89.907	36.461	17.153	5.716	0.694	–
	Town	91.897	38.271	9.611	6.538	2.938	0.209
2010	City	45.318	73.306	21.072	10.231		
	County	66.573	57.932	13.061	9.516	2.786	0.104
	Town	78.474	48.839	10.285	6.390	4.663	0.813
2015	City	104.923	45.008	–	–	–	–
	County	109.028	39.313	1.590	–	–	–
	Town	112.505	31.600	4.845	0.483	0.029	0.002
2020	City	55.675	78.066	16.190	–	–	–
	County	75.047	57.125	11.773	5.604	0.382	–
	Town	83.636	47.321	10.956	5.265	2.081	0.174

It can be seen from Fig. 11 that at the city scale, the high erosion grade regions are mainly concentrated in mountainous areas. On the county scale, the dispersion trend of areas with higher erosion intensity grades is gradually significant. Compared with the city scale, some areas with high erosion intensity grades are significantly increased, including some municipal districts in Zibo, some municipal districts in Jinan, and a few municipal districts in Hebi. From the perspective of the distribution on the town scale, the patches with higher grades are mostly focused in the surrounding areas of Jinan, Anyang, Zhengzhou and Zhoukou, showing a more dispersed and specific distribution. At the micro scale, the serious soil erosion areas are more clearly exposed to the administrative jurisdiction, which is convenient for the management of administrative divisions. In general, with the reduction of the scale, the higher-grade soil erosion area is gradually increasing, and the distribution is more and more detailed, which is beneficial to put forward and formulate soil conservation measures on a small scale based on regions of serious erosion, and effectively prevent and control erosion in AALYR from a micro perspective.

### 3.3 Analysis of influencing factors based on geodetector

Soil erosion process is complex. To explore the differentiation of soil erosion, we select six factors of land use type, elevation, slope, NDVI, annual precipitation and population for analysis with 2020 as the base year. The single factor and interactive detection results of AALYR by Geodetector are shown in Table 7, Table 8.

**Table 7:** Detection of soil erosion influence factors.

Variable	Land use	Elevation	Slop	NDVI	Annual precipitation	Population
q value	0.0563	0.1192	0.0405	0.0270	0.0416	0.0130
P value	0.000	0.000	0.000	0.000	0.000	0.000

**Table 8:** Interactive detection results.

	Land use	Elevation	Slop	NDVI	Annual precipitation
Elevation	0.1917				
Slop	0.1260	0.2419			
NDVI	0.1067	0.2350	0.1462		
Annual precipitation	0.1219	0.2329	0.1902	0.1322	
Population	0.1379	0.1945	0.1206	0.1111	0.1412

The q reflects the influence of factors (Table 7). From Table 7, The factors are ranked of influence as follows: elevation > land use type > annual precipitation > slope > NDVI > population, all of which passed the significance test (P < 0.05). Among them, elevation (11.92%) and land use type (5.63%) have strong explanatory power.

The interactive detection reflects the influence of the interaction between the factors on soil erosion (Table 8). Table 8 reflects that soil erosion is a complex process and indicating that the interaction between factors in this paper has different degrees of enhancement in the explanatory power of soil erosion compared with single factor. Among them, the interaction between elevation and slope has the largest explanatory power, which is 24.19%, followed by the interaction between elevation and NDVI, which is 23.50%, and the interaction between elevation and annual precipitation is 23.29%, indicating that elevation and slop during this period are important factors leading to soil erosion.

**Fig. 12:**
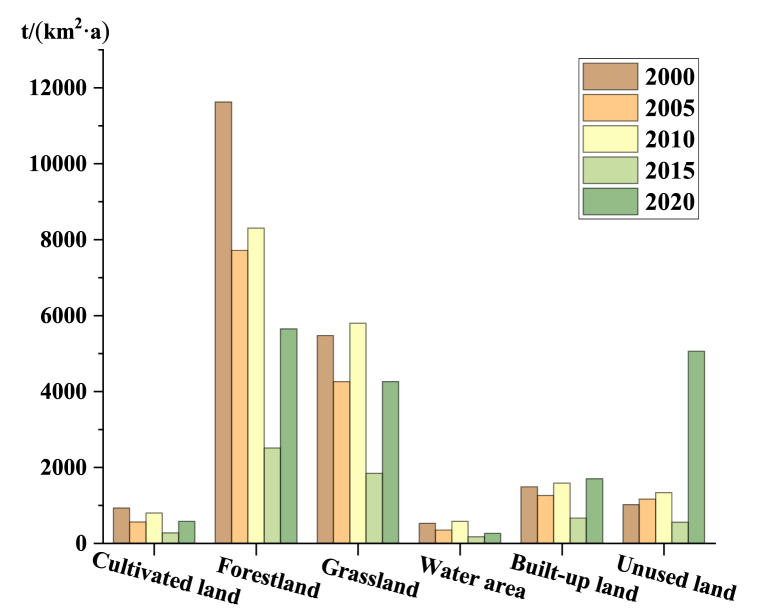
Soil erosion amount based on land use type (t/(km^2^·a)).

The above detection results indicate that except topographic, the main factor affecting soil erosion is land use. Soil erosion of different land use type is calculated by ArcGIS 10.3, as shown in Fig. 12. Fig. 12 shows that there is variation in soil erosion per unit area for different types of land during study period. On the whole, the largest of soil erosion by land use type is forestland. In terms of time, the soil erosion of forestland and grassland water area show a trend of “increase-decrease-increase”, the cultivated land and water show a trend of decreasing fluctuation. Of these, soil erosion was relatively lightest in 2015.

## 4 Discussion

The soil erosion in AALYR from 2000 to 2020 is estimated by RUSLE, and the results show that the erosion grade is mainly very slight, and the severe and above erosion grades are mainly distributed in Zibo, Jinan, Anyang, Zhengzhou and Tai 'an. The common feature of these cities with serious erosion is that the elevation is relatively high. According to the RUSLE model, *K* and *LS* don’t change much, *C* depends on the surface vegetation coverage and land use type has a great relationship, *P* value is obtained directly according to the land use type assignment. So, the soil erosion is mainly affected by rainfall and land use. *R* of the whole study area in 2000–2020 shows a trend of “decline-rise-decline-rise”. Among them, it is particularly obvious that the rainfall erosivity is very small in 2015 due to the influence of drought, and the maximum value don’t reach one third of the maximum value in 2005, and the annual average erosion amount in this year is also the smallest due to the rainfall erosivity, which shows the impact of rainfall. Soil erosion is influenced to some extent by the rainfall erosion factor, but it is not entirely dependent on the change of rainfall but is also affected by other factors. Additionally, when we research the spatiotemporal evolution characteristics of erosion grades and find that the percent of slight and below area is mostly about 85%. While the area percentage of the same grade in Henan Province researched by Liu et al. [59] is 88.39%, and in the study of mountainous areas of south-central Shandong by Li et al. [60], the proportion of the same grade of area is also about 85%, which reflected the reliability of the conclusions of this study. Then we find that the smaller the scale, the more detailed the serious soil erosion area, which is beneficial to formulate soil and water conservation planning for severe areas with administrative divisions as management units.

Thereafter, the results of influencing factors analyzing show that elevation is the main impact factor of soil erosion, followed by land use type. Fig. 8 also shows that the area with high elevation has serious erosion. Among the different types of land, forestland and grassland have a high degree of erosion, which is affected by vegetation coverage and land use intensity of various types of land. In the studies about YRB [61,62], soil erosion in YRB is declining, in which topography is the important influencing factors, and our conclusion is the same as that. Different from their research, we only analyzed the influencing factors in 2020 and lacked the comparative analysis of the changes of the main influencing factors in each period. After that, we can go deep into this aspect of the research. The effect of land use is actually the influence of human activity-induced changes in land use types on soil erosion. Previous studies [63,64] pointed out that human activities have a greater impact than climate change. We should strengthen the management of soil erosion. In fact, since 1999, return to forestry measures have played an essential role in controlling soil erosion as evidenced by the increase in vegetation cover [65,66], whose role in soil erosion control is very important.

AALYR is the key development area of YRB. Previous studies about erosion in AALYR are relatively lacking. And this research is important for the ecological civilization construction and sustainable development of AALYR. The selection of the calculation data of each factor in this paper is consistent with Yang et al. [67] (*R* factor), Qian et al. [19] (*K* factor), Guo et al. [68] (*P* factor), Yin et al. [61] (*LS* factor), Qian et al. [69] (*C* factor), which reflects the scientific nature of the estimation in this paper. The social activities in AALYR are complex and diverse, the land use change is a long-term and slow process. Because of the limitation of data sources, the data of AALYR in 2000, 2005, 2010, 2015 and 2020 are selected. The accuracy of the model calculation is affected by the accuracy of the input data. When calculating the RUSLE model, due to the difficulty of obtaining high-precision data, a simple algorithm is selected to determine the calculation method of the *R* factor, which would cause the deviation of the results to a certain extent.

To summarize, in future studies, data accuracy should be improved as much as possible, and the calculation of each factor should be optimized to promote the precision of the results. In addition, RUSLE has a simple structure, fewer parameters, and more accurate predictions of average soil erosion compared with other soil erosion models [70]. However, there are some models and methods for quantitative estimation of soil erosion, such as WEPP model, InVEST model, WaTEM/SEDEM model, CSLE model and so on. Among them, CLSE is a soil erosion estimation method derived by Chinese scholars on the basis of soil loss equation, which considers the China’s characteristics of soil erosion. In the future, we can update the data source, refine the research period, try a variety of methods according to the research focus, and carry out deeper research and analysis from more relevant influencing factors.

## 5 Conclusions

With the increasing impact of global warming and human disturbance, soil erosion has become a serious and lasting environmental challenge affecting ecological construction and social economic development [71]. The high-quality development of AALYR has very important strategic significance for the development of China. Conclusions in the text can serve as foundations for developing reasonable soil and water conservation measures in AALYR. What’s more, it has important practical significance for local agriculture, industry, economic production, and environmental protection.

The analysis revealed that although soil erosion in AALYR varies from 2000 to 2020, it shows a general pattern of “northeast high, middle low, southwest high”. Annual average soil erosion shows the most obvious downward trend from 2010 to 2015. Overall, the erosion grade is changing to very slight and slight grade, and the soil erosion status is also significantly improved. When analyzing the erosion characteristics at the city, county, and town scales, it is concluded that with the reduction of the scale, the area of serious erosion area gradually increases and the distribution gradually disperses, but the location characteristics of serious erosion area will be more detailed. In the final influencing factors analysis, soil erosion is mainly affected by elevation and land use. Soil erosion in high altitude areas is more serious than that in low altitude areas. Forestland is the land type with the highest soil erosion, followed by grassland.

Generally, AALYR belongs to the cultivation area, human activities are frequent, and the built-up land is gradually expanding, which will greatly increase the soil erosion, so the ecological protection of soil should be paid attention to. Based on this, we should vigorously promote the advantages of protecting the soil environment. Depending on the erosion condition in AALYR, scientific planning and rational use of land; returning large slope cultivated land to forestland, changing unreasonable cultivation system; increasing organic fertilizer, rational rotation, and improving soil erosion resistance. Create water conservation forests near the reservoir area and the lake area to prevent silt accumulation; through small water conservancy projects to protect and rationally use water and soil resources to prevent soil erosion and restore the ecological environment.

## Data availability statement

Data will be made available on request.

## CRediT authorship contribution statement

**Ying Zhang:** Writing – original draft, Methodology. **Pengyan Zhang:** Writing – review & editing, Project administration, Funding acquisition. **Zhenyue Liu:** Software. **Guangrui Xing:** Software. **Zhuo Chen:** Writing – review & editing. **Yinghui Chang:** Investigation. **Qianxu Wang:** Investigation.

## Declaration of competing interest

The authors declare that they have no known competing financial interests or personal relationships that could have appeared to influence the work reported in this paper.
