# Supplementary material for: Dynamic analysis of soil erosion in the affected area of the lower Yellow River based on RUSLE model
Source: Heliyon. 2023 Dec 20;10(1):e23819. doi: 10.1016/j.heliyon.2023.e23819 (PMC10788514; doi:10.1016/j.heliyon.2023.e23819)
Supplement: Multimedia component 1 [file mmc1.docx]

Description of soil properties：

| Particle-size class | Diameter of particles |
| --- | --- |
| Sand | > 63 µm - ≤ 2 mm |
| Silt | > 2 µm - ≤ 63 µm |
| Clay | ≤ 2 µm |

From IUSS Working Group WRB. 2022. World Reference Base for Soil Resources. International soil classification system for naming soils and creating legends for soil maps. 4th edition. International Union of Soil Sciences (IUSS), Vienna, Austria, 217 p.
